# Kinetic Spectrophotometric Determination of Biotin in Pharmaceutical Preparations

**Published:** 2008-09

**Authors:** M. I. Walash, M. Rizk, Z. A. Sheribah, M. M. Salim

**Affiliations:** *Analytical Chemistry Department, Faculty of Pharmacy, Mansoura University, Mansoura, Egypt*

**Keywords:** biotin, sodium azide, tri-iodide, kinetic, pharmaceutical preparations

## Abstract

A simple accurate kinetic spectrophotometric method was developed for the determination of biotin in pure form and pharmaceutical preparations. The proposed method is based on a catalytic acceleration of biotin on the reaction between sodium azide and tri-iodide in an aqueous solution. Concentration range of 4-16 μg/mL for biotin was determined by measuring the decrease in the absorbance of tri-iodide at 348 nm by a fixed time method. The decrease in absorbance after 14 min from the initiation of the reaction was markedly correlated to the concentration with correlation coefficient of 0.9999. The detection limit (LOD) of biotin was 0.18 μg/mL while quantitation limit (LOQ) was 0.54 μg/mL. The percentage recovery of the spiked samples was 100.08 ± 0.761. The proposed procedure was successfully applied for the determination of biotin in its pharmaceutical preparations with mean recoveries of 101.17 ± 2.05 and 97.87 ± 1.50 for biotin ampoules and capsules, respectively. The results obtained were in good agreement with those obtained using official method.

## INTRODUCTION

**Figure 1:**
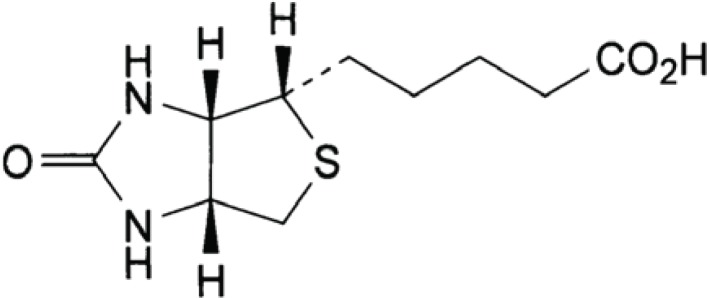
Structural formula of biotin.

Biotin, Hexahydro-2-oxo-1H- thieno (3,4-d) imidazole -4-pentanoic acid (Fig. 1), is also known as vitamin H or Co-enzyme R (1). It is a water-soluble vitamin belonging to the B-complex. It acts as a cofactor responsible for the carbon dioxide transfer in several carboxylase enzymes. It is involved in the biosynthesis of fatty acids, gluconeogenesis, and energy production, the metabolism of the branched chain amino acids and the De Novo synthesis of purine nucleotides. Recent research indicates that biotin plays a role in gene expression and that it may act in DNA replication (2).

The United States Pharmacopoeia (USP) (3); recommends an acid-base titration of biotin with 0.1 N NaOH using phenolphthalein as indicator. On the other hand, the British Pharmacopoeia (BP) (4); reports a non-aqueous potentiometric titration of the studied drug with 0.1 M tetrabutylammonium hydroxide.

Various methods have been published for determination of biotin either *per se* or in pharmaceutical preparations including: spectrophotometry (5, 6), spectrofluorometry (7), electro-analysis (8-10) and High Performance Liquid Chromatography (HPLC) (11-17), Liquid Chromatography-Mass Spectrometry (LC-MS) (18, 19), capillary zone electrophoresis (20, 21), Microbiological methods (22), Bioassays (23), Radioisotopic binding assays (24-26) and Non-Radioisotopic binding assays (27-32).

Biotin does not manifest ultra-violet absorption spectrum for its detection (33). UV detection which is mainly employed for HPLC suffers from the low absorbance above 210 nm. This is a problem because most of the conventionally used solvents strongly absorb radiation themselves above 210 nm. Therefore, derivative and complex formation is necessary in most cases (21).

Regarding the literature review, the spectrophotometric methods which were reported for the determination of biotin, based on measurement of absorbance of tri-iodide/chloroform extract at 520 nm after oxidation with potassium iodate (5), and measurement of absorbance of tri-iodide at 350-352 nm produced after reaction with periodate (6), however, the sensitivity was rather poor, the working range was 3-18 mg/g and 20-80 μg/ml respectively. Up till now no kinetic spectrophotometric method has been reported for determination of biotin. This initiated our present study.

The catalytic kinetic spectrophotometric method is one of the most attractive approaches for determination of certain compounds and has many advantages:

- Selectivity due to the measurement of the evolution of the absorbance with the time of reaction instead of the measure of a concrete absorbance value;
- Possibility of no interference of the colored and/or turbidity background of the samples;
- Possibility of no interference of other active compounds present in the commercial product. (34).

The aim of the present work was to establish a simple kinetic spectrophotometric method for determination of biotin in certain pharmaceutical dosage forms, based on the catalytic effect of biotin on the tri-iodide-azide reaction which has been widely applied to the determination of compounds viz, captopril and ethamsylate (35), carbocisteine, ethionamide, thioctic acid and penicillamine (36) and glutathione and tetramethylthiuram disulphide (37).

## EXPERIMENTAL

### Apparatus

A Shimadzu (Model 1601 PC) UV-Visible spectrophotometer (Shimadzu, Kyoto, Japan) was used to measure the absorbance at 348 nm. The cell chamber was kept at a specified temperature using a Shimadzu Thermostat Model T.C.C. Controller.

### Reagents and materials

All reagents were of analytical grade and the water was always double distilled water.

- Biotin was kindly provided from Pharco Pharmaceuticals (Alex. - Egypt), its purity was 99.02 % which determined by applying official method (3);
- Tri-iodide aqueous solution (0.01 M) was prepared by dissolving 0.254 g of iodine in 100 mL of distilled water containing 4.5 g of potassium iodide and further diluted with the same solvent to obtain (0.001 M) concentration;
- Sodium azide solution (1.0 M) was prepared by dissolving 6.50 g of sodium azide in 100 ml of distilled water;
- Phosphate buffer solutions (pH2-10), were prepared using 0.1 M Na_2_HPO_4_ and 0.1 M NaOH (4);
- HCl aqueous solution (0.1 M); pH 1;
- Sodium hydroxide (0.1 M) aqueous solution, (El-Nasr Pharmaceutical Chemicals (ADWIC), Egypt);
- Stock solutions.

A stock solution of biotin was prepared by dissolving 100.0 mg of biotin in 1 mL of 0.1 M NaOH and then was completed to 100 ml with distilled water. Working standard solutions were prepared by dilution with distilled water. The standard solutions were found to be stable for one week when kept in the refrigerator at 4°C.

### Procedures

**Construction of calibration graphs.** Accurately measured aliquots of biotin working standard solution covering the working concentration range from 4-16 μg/mL, were transferred into a series of 10 mL volumetric flasks. 1 ml phosphate buffer of pH 4 was added followed by 1 mL (1.0 M) of sodium azide solution. The solutions were mixed well and diluted to 8 ml with distilled water. The solutions were kept for 3 min. Then 1ml of 0.001 M tri-iodide solution was added and the volume was adjusted to the mark with distilled water. An aliquot of the reaction solution was quickly transferred into a quartz cell within 40 sec, and then it was placed in a chamber of spectrophotometer (kept at 25°C). The absorbance of the remaining unreacted tri-iodide (A) was recorded at 348 nm *versus* time for 14 min. The absorbance of blank experiment (A_0_) was performed simultaneously. The difference in absorbance (ΔA) was plotted *versus* the drug concentrations (μg/mL) to get the calibration graph; alternatively, the regression equation was derived.

**Procedure for the pharmaceutical preparations.** An accurately weighed quantity of the mixed contents of 10 capsules or accurately measured volume of the injection solution equivalent to 10.0 mg of the drug transferred into a small conical flask and extracted or diluted with 3 × 30 ml of distilled water, respectively. One milliliter of 0.1 M NaOH was firstly added. The extract was sonicated for 15 minutes and filtered if necessary into 100 ml volumetric flask. The conical flask was washed with several mLs of distilled water. The washings were passed into the same volumetric flask and completed to the mark with the same solvent. The above procedure was then followed. The nominal content was calculated either from the previously plotted calibration graph or using the corresponding regression equation.

## RESULTS AND DISCUSSION

Iodine oxidizes sodium azide in acid medium to form iodide and nitrogen.

${2N}_{3}^{-}+I_{2}\to {3N}_{2}+{2I}^{-}$

This reaction is immeasurably slow in the absence of catalysts (38).

${\mathrm{RS}}^{-}+I_{2}\to \mathrm{RSI}+I^{-}$

$\mathrm{RSI}+{2N}_{3}^{-}\to {\mathrm{RS}}^{-}+I^{-}+{3N}_{2}$

**Figure 2:**
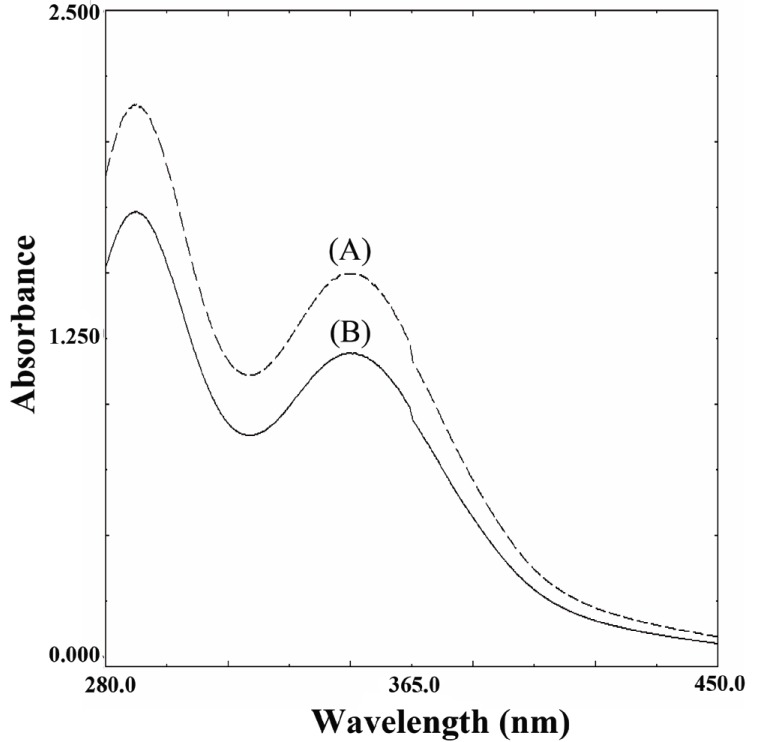
Absorption spectra: (A) Blank; (B) catalytic effect of biotin (5 μg/mL) on the reaction using 1 mL (1.0 M) sodium azide solution and 1 mL (0.001 M) tri-iodide aqueous solution.

This reaction is very much accelerated in the presence of sulfide or thiol containing compounds. This reaction can be followed spectrophotometrically by monitoring the decrease in the absorbance of tri-iodide at 348 nm as shown in Figure 2.

### Optimization of the reaction conditions

**Effect of reaction time.** There are many methods, such as fixed time, initial rate and rate constant, for measuring the catalytic effects among them, the fixed time measurement is the most conventional and simplest. The most suitable reaction time was found to be 14 min after the addition of tri-iodide for biotin based on its correlation coefficient of the calibration curve as shown in Table 1.

**Table 1:** Feasibility of the fixed time method for the determination of biotin by the proposed method

Time (min.)	Regression Equations	Correlation Coefficient

2	A= -0.029 + 7.7 × 10^-3^C	r=0.9670
4	A= -0.022 + 0.012C	r=0.9870
6	A=-0.029 + 0.016C	r=0.9950
8	A= -0.025 + 0.019C	r=0.9950
10	A= -0.012 + 0.021C	r=0.9970
12	A= -0.018 + 0.024C	r=0.9997
14	A= -0.024 +0.027C	r=0.9999
15	A= -0.023 + 0.026C	r=0.9999

**Figure 3:**
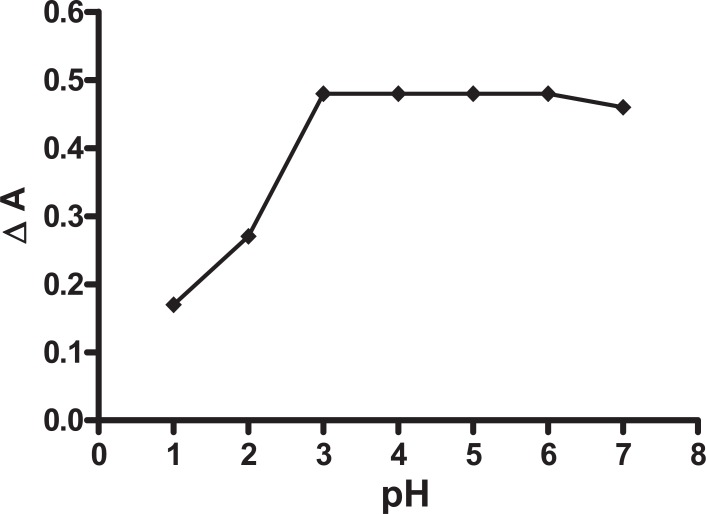
Effect of pH on the reaction between biotin (20 μg/mL) and 1 mL (0.001 M) tri-iodide aqueous solution, 1 mL (1.0 M) sodium azide at Δt=14 min.

**Effect of pH.** The effect of pH on the catalyzed (sample) and un-catalyzed (blank) reactions was studied, using 1 mL of 0.001 M tri-iodide solution and 1 mL of 1.0 M azide solution. The maximum difference in absorbance (ΔA) was observed at phosphate buffer of pH 4 (Fig. 3). Other buffers having the same pH value such as acetate and citrate were studied and compared with phosphate buffer. Phosphate buffer was proved to be superior over acetate and citrate buffers as revealed by high sensitivity.

**Figure 4:**
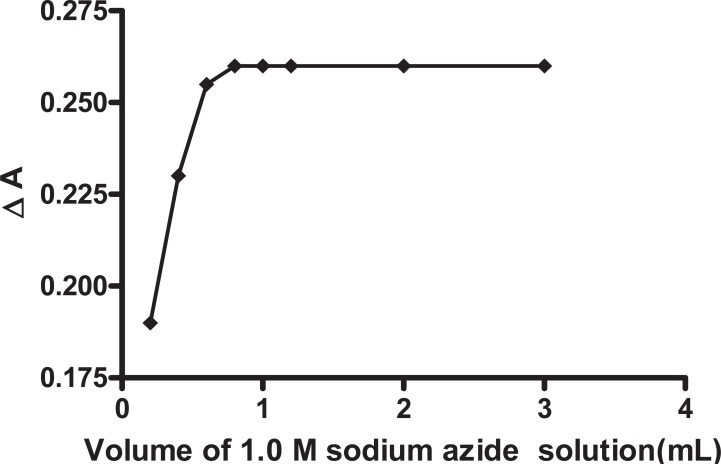
Effect of sodium azide volume on the reaction between biotin (10 μg/mL) and 1 mL (0.001 M) tri-iodide aqueous solution Δt=14 min.

**Effect of azide concentration.** It was found that 1 mL (1.0 M) of sodium azide solution was sufficient to give a maximum absorbance after which the absorbance value remains constant (Fig. 4). Thus 1 mL (1.0 M) of sodium azide solution was used as optimum volume through this approach.

**Effect of tri-iodide concentration.** As the volume of 0.001 M tri-iodide solution increased, the net reaction rate increased. But the volumes larger than 1 mL affect the reproducibility of the proposed method due to the excessive brown colour background; so, 1 mL of 0.001 M tri-iodide is used.

**Effect of temperature.** The effect of temperature was studied in the range of (5-60°C). As the temperature increased, the rate of both catalyzed and un-catalyzed reactions increased up to 50°C for biotin. Above this temperature, ΔA values decreased which suggested that the rate of un-catalyzed reaction was also accelerated, or iodine became lost due to volatilization upon heating. It was found that 25°C for 14 min after addition of iodine solution has more reproducible catalytic effect.

### Validation of the Method

**Accuracy and precision.** The proposed method was evaluated by calculating the accuracy as percent relative error (% Error) and precision as percent relative standard deviation (RSD%). The data obtained are abridged in Table 2.

**Table 2:** Performance data of the proposed method

Parameter	Biotin

Concentration range (μg/mL)	4-16
Limit of detection (LOD) (μg/mL)	0.18
Limit of quantification (LOQ) (μg/mL)	0.54
Correlation coefficient(r)	0.9999
Slope	0.0279
Intercept	1.084 × 10^-3^
Standard deviation of the residuals, S_y/x_	1.689 × 10^-3^
Standard deviation of the intercept, S_a_	1.490 × 10^-3^
Standard deviation of the slope, S_b_	0.146 × 10^-3^
Relative standard deviation, % RSD	0.761
Percentage error, % Error	0.269

Statistical analysis (40) of the results, obtained by the proposed and the official method (3) using Student’s t-test and variance ratio F-test, shows no significant difference between the performance of the two methods regarding the accuracy and precision, respectively (Table 3). The official method involved acid-base titration of biotin using 0.1 N NaOH as a titrant and phenolphthalein as indicator (3).

- **Repeatability:** The repeatability and reproducibility (intra-day precision) was evaluated through replicate analysis of biotin authentic sample spiked with 8.0 μg/mL on three successive times on the same day. The mean percentage recoveries based on the average of three separate determinations were 100.89 ± 1.33 as shown in Table 4.
- **Intermediate precision:** The Intermediate precision (inter-day precision) was evaluated through replicate analysis of Biotin spiked with 12.0 μg/mL on three successive days. The percentage recoveries based on the average of three separate determinations were 99.50 ± 4.56 (Table 4).

**Table 3:** Application of the proposed and official methods to the determination of biotin in pure form

Compound	Proposed method			Official method (3)
	Concentration taken (μg/mL)	Concentration found (μg/mL)	Recovery (%)	Recovery (%)

Biotin	4.0	3.976	99.40	98.04
	5.0	4.979	99.58	100.98
	6.0	6.090	101.50	98.04
	8.0	8.026	100.33	
	10.0	9.997	99.97	
	12.0	11.968	99.73	
	14.0	13.940	99.57	
	16.0	16.090	100.56	
Mean ± S.D			100.08 ± 0.761	99.02 ± 1.69
Student’s t-test			1.42 (2.26)	
F-test			5.95 (19.35)	

Each result is the average of three separate determinations. Values in parentheses are the tabulated t and F values, respectively at *p*=0.05 (40).

**Table 4:** Validation data of the proposed method for the determination of biotin in pure form

Regimen	Parameters	Recovery %		
		Amount Added (μg/mL)	Amount Found (μg/mL)	Recovery %

Intra-day		8.0	8.071	100.89
		8.0	8.178	102.22
		8.0	7.965	99.56
	(x)			100.89
	±S.D.			1.33
	% RSD			1.318
	% Error			0.767
Inter-day	1^st^ day	12.0	11.821	98.51
	2^nd^ day	12.0	11.462	95.52
	3^rd^ day	12.0	12.538	104.48
	(x)			99.50
	±S.D.			4.561
	% RSD			4.584
	% Error			2.633

### Concentration ranges and calibration graphs

After optimizing the reaction conditions, linear calibration graph was obtained over the range of 4-16 μg/mL. Analysis of the data gave the following regression equation:

ΔA=1.08×10−3+0.0279 C  r=0.9999

**Figure 5:**
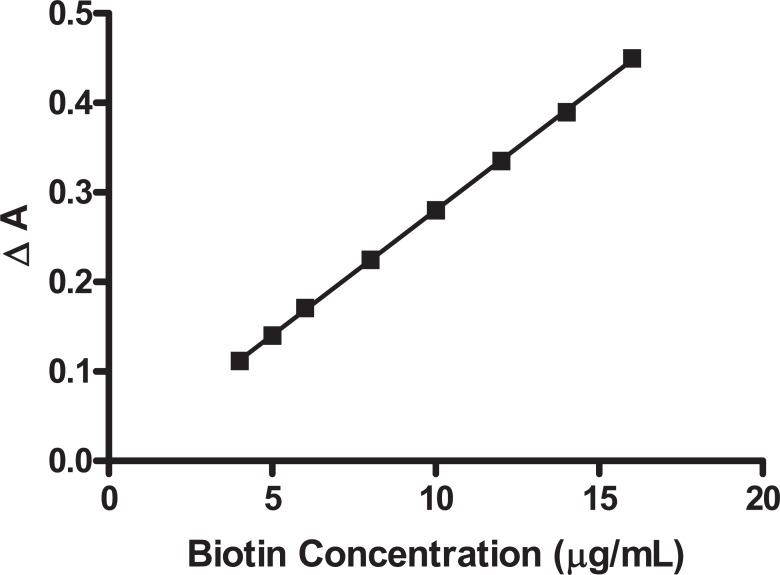
Calibration graph of biotin determined by the proposed method.

Where is ΔA the difference in absorbance between blank and drug, C is the concentration of the drug in μg/mL and r is the correlation coefficient. The calibration graph adopting the proposed method is shown in Figure 5.

Validation of the method was evaluated by statistical analysis (40) of the regression data regarding standard deviation of the residuals (S_y/x_), the standard deviation of intercept (S_a_), and standard deviation of the slope (S_b_). The data obtained are included in Table 2. The small values given point out to the low scattering of the points of the calibration curve.

### Limit of quantitation and limit of detection

The limit of quantitation (LOQ) was determined by establishing the lowest concentration that can be measured according to ICH Q2 (R1) recommendation (39) below which the calibration graph is non linear and was found to be 0.54 μg/mL.

The limit of detection (LOD) was determined by evaluating the lowest concentration of the analyte that can be readily detected and was found to be 0.18 μg/mL. LOQ and LOD were calculated according to the following equations (39):

LOQ = 10 S_a_/b

LOD = 3.3 S_a_/b

Where S_a_ is the standard deviation of the intercept of regression line, and b is the slope of the calibration curve.

### Robustness of the method

The robustness of the proposed method is demonstrated by the constancy of the ΔA with the minor changes in the experimental parameters such as pH 4 ± 0.2 and sodium azide (1.0 M) volume, (1 mL ± 0. 5). These minor changes that may take place during the experimental operation didn’t greatly affect the absorbance difference (ΔA).

### Pharmaceutical Applications

The proposed method was applied to the determination of biotin in its dosage forms. The selectivity of the method was investigated by observing any interference encountered from the capsule excepients. These excepients did not interfere with the proposed method (Table 5). The results of the proposed method were statistically compared with those obtained using the official method. The latter method involved HPLC determination of biotin in its dosage forms. The liquid chromatograph is equipped with a 200 nm detector and a 4.6 mm × 15 cm column containing 3 μm packing L7. The flow rate is about 1.2 mL per minute (3).

**Table 5:** Application of the proposed method to the determination of biotin in its pharmaceutical preparations

Compound	Proposed method			Official method (3)
	Concentration taken (μg/mL)	Concentration found (μg/mL)	Recovery (%)	Recovery (%)

**Biotine Bayer 0.5% inj**^a^	8.0	8.200	102.50	102.09
(Biotin 5 mg/mL)	10.0	10.222	102.22	99.98
Batch # F0120	12.0	11.857	98.81	100.70
Mean ± S. D			101.17 ± 2.05	100.92 ± 1.07
% RSD			2.030	1.062
%Error			1.172	0.613
Student’s t-test			0.11 (2.78)	
F-test			3.67 (19.00)	
**Biotin forte capsules**^b^	6.0	5.965	99.42	98.96
(Biotin 5 mg/capsule)	8.0	7.822	97.78	99.93
Batch # 7433017	12.0	11.570	96.42	99.45
Mean ± S. D			97.87 ± 1.50	99.44 ± 0.48
% RSD			1.534	0.487
%Error			0.886	0.281
Student’s t-test			1.73 (2.78)	
F-test			9.59 (19.00)	

Each result is the average of three separate determinations. Values in parentheses are the tabulated t and F values, respectively at *p*=0.05 (40).

a Product of Bayer Sante Familiale, Les Moulineaux Cedex 9, France;

b Product of Unipharma, El Obour City, Cairo, Egypt.

Statistical analysis (40) of the results obtained using Student’s t-test and variance ratio F-test revealed no significant difference between the performance of the two methods regarding the accuracy and precision, respectively (Table 5).

## CONCLUSION

The results suggested that the observed decrease in the absorbance at 348 nm was mainly due to the catalytic effect of biotin on the reaction between iodine and azide, rather than a direct reaction between tri-iodide compounds. In conclusion, the proposed method was accurate, precise, sensitive, rapid, low cost, relatively selective and was successfully utilized in determination of biotin in its different dosage forms.
